# Boosting Water Retention in Agriculture: Vine Biochar‐Doped Hydrogels' Swelling and Germination Effects

**DOI:** 10.1002/gch2.202300254

**Published:** 2024-03-29

**Authors:** Yağmur Uysal, Zeynep Görkem Doğaroğlu, Mehmet Nuri Makas, Zehranur Çaylali

**Affiliations:** ^1^ Mersin University Engineering Faculty Environmental Engineering Department Mersin 33343 Turkey

**Keywords:** biochar‐doped hydrogels, swelling, vine pruning waste, water sorption, wheat seeds

## Abstract

Water scarcity presents a formidable challenge to agriculture, particularly in arid, semiarid, and rainfed settings. In agricultural contexts, hydrogels serve as granular agents for water retention, undergoing considerable expansion upon water exposure. They assume versatile roles encompassing soil‐water retention, the dispensation of nutrients and pesticides, seed encapsulation, erosion mitigation, and even food supplementation. This study's objective involves the examination of biochar‐infused hydrogels, fashioned by incorporating vine pruning waste‐derived biochars, and the assessment of swelling behaviors in various aqueous environments encompassing deionized, tap, and saline water at concentrations of 0.5–1%. Characterizations of the vine‐biochars‐VB and biochar‐incorporated hydrogels‐VBHG are executed, with particular attention to their swelling properties across diverse media. As an initial step toward appraising their agricultural relevance, these hydrogels are introduced to a germination medium featuring wheat seeds to discern potential influences on germination dynamics. The maximum swelling capacity of VBHG is recorded in deionized water, tap water at pH 7.0, tap water at pH 9.0, saline water at 0.5%, and saline water at 1%, reaching 352%, 207%, 230%, 522%, and 549%, respectively. Remarkably, the 0.5% VBHG treatment exhibits the most pronounced root elongation. The application of hydrogels in agriculture exhibits promise, particularly within drought‐related contexts and potential soilless applications.

## Graphical abstract

This study presents biochar‐doped hydrogels studied for wheat seed germination using vine pruning biochar. The use of biomass waste in hydrogel production promotes sustainable agriculture. Biomass‐doped hydrogels show promise in enhancing seed germination and crop growth through enhanced water retention.

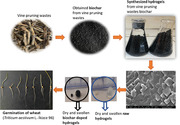

## 1 Introduction

Water scarcity severely threatens agriculture in arid and semiarid regions due to low rainfall and irregular distribution. Deficit irrigation technologies are essential for managing restricted water supply, maintaining favorable soil moisture for root zones, and improving water use efficiency without compromising crop yield and quality.^[^ ^1^, ^2^ ^]^ However, these technologies are costly and require expertise, primarily benefiting high‐value crops. Hydrogel polymer technology's use has surged as a multifunctional soil conditioner, addressing water absorption and retention in arid climates.^[^ ^3^, ^4^, ^5^ ^]^ By curbing evaporation, deep percolation, and nutrient leaching, these polymers sustain high water‐swelling and moisture‐releasing capacities under water‐deficient conditions, enhancing plant growth and crop yield.

Hydrophilic polymers create hydrogels, absorbing and retaining water in 3D structures.^[^ ^6^ ^]^ The network forms via chemical crosslinking,^[^ ^7^ ^]^ physical chain entanglement,^[^ ^8^ ^]^ hydrogen bonds,^[^ ^9^ ^]^ or ionic bonds.^[^ ^10^ ^]^ Hydrogel properties depend on polymer type, composition, and manufacturing. Crosslinking defines its nature – physical (hydrophobic, chain, hydrogen), chemical (covalent), or dual‐network.^[^ ^11^ ^]^ Various sources contribute to hydrogel fabrication – synthetic polymers like pHEMA, polyacrylamide, polyvinyl alcohol, and derivatives are prevalent, offering enhanced strength and water retention.^[^ ^12^ ^]^ Composite hydrogels result from physical/chemical processes (crosslinking, grafting, blending), yielding novel characteristics and uses.^[^ ^13^ ^]^ Bio‐based hydrogels hold promise in biomedicine, environment, and agriculture, contingent on water sorption, stability, and strength. Natural materials (starch, chitosan, dextran, alginate) are investigated for chemical hydrogel production in tissue engineering and environmental applications due to their biocompatibility, non‐toxicity, and gel‐forming properties. The versatility of these materials opens avenues for innovative solutions to challenges across diverse fields.

Biochar (BC) is a carbon‐rich, porous material formed by biomass pyrolysis at 350–900 °C under anoxic conditions.^[^ ^14^ ^]^ It's produced through various methods like gasification, hydrothermal carbonization, quick, and slow pyrolysis.^[^ ^15^ ^]^ Pyrolysis conditions and the type of raw material affect the chemical structure of the product.^[^ ^16^, ^17^ ^]^ The main reason why these products differ in terms of values ​​such as nitrogen, sulfur, calcium, magnesium, and phosphorus concentrations, larger surface area, and stronger cation exchange capacities, carbon and ash content is primarily the pyrolysis temperature, as well as the type of raw material and total nutritional content.^[^ ^18^, ^19^ ^]^ Slow pyrolysis eliminates volatile components like oxygen, hydrogen, nitrogen, total phosphorus, and sulfur.^[^ ^20^ ^]^ Pyrolyzed biochar effectively immobilizes heavy metals in the soil through methods like ion exchange, co‐precipitation, and adsorption.^[^ ^21^ ^]^ Industrial‐scale pyrolysis with low oxygen produces carbonaceous material.^[^ ^22^ ^]^ Biochar has applications in construction, animal husbandry, composting, soil remediation, energy storage, and agronomy.^[^ ^23^ ^]^ Its diverse uses reflect its potential to address challenges across various fields.

Biochar (BC) holds potential as a soil conditioner due to agronomic, financial, and environmental benefits.^[^ ^24^ ^]^ Around 40–75% of BC's carbon comprises complex organic matter, challenging for microbes to decompose.^[^ ^25^ ^]^ Biochar contains functional groups and a substantial surface area,^[^ ^26^ ^]^ but using pure BC as a soil amendment has drawbacks: low adsorption capacity, struggles with specific pollutants, and mobility of heavy metals.^[^ ^27^ ^]^ To enhance its environmental use, BC needs functionalization into a composite. The hydrogel‐biochar composite absorbs mineral nutrients in its cross‐linked structure, securely retaining them and delaying decomposition.^[^ ^28^ ^]^ Its cost‐effectiveness, stemming from industrial, animal, and agricultural waste sources, further boosts its appeal.^[^ ^29^ ^]^

Hydrogel‐based soil additives gain attention for water retention and soil stability.^[^ ^30^ ^]^ Combining hydrogel with biochar forms a promising soil additive, enhancing nitrogen use and immobilizing heavy metals. Limited studies explore the hydrogel‐biochar combination for soil improvement. Organic matter like vineyard pruning waste benefits soil structure, nutrients, and chemistry.^[^ ^31^ ^]^ In this study, vineyard pruning wastes from Mersin, Turkey, were used to create biochar. Biochar‐doped hydrogels were synthesized and characterized. The kinetics of swelling, water retention in aqueous and soil environments, and other properties of these hydrogels were examined.

The objective of this study is to synthesize a biochar‐hydrogel composite in a cost‐effective manner. The composite, when applied in agriculture, is expected to improve water retention in soil compared to non‐biochar composites. The biochar component is anticipated to enhance water retention. Varying biochar amounts will impact the composite's structure. Vine pruning waste‐derived biochar and hydrogel composites (VBHG) were produced for this investigation. Our study innovatively utilizes carbon, nitrogen, and phosphorus from pruning waste to create valuable biochars and convert them into functional hydrogels. We characterized synthesized biochars (VB), biochar‐doped hydrogels (VBHG), and raw hydrogels through techniques like Fourier Transform Infrared Spectroscopy (FT‐IR) and Scanning Electron Microscopy (SEM). We assessed the swelling capacities of biochar/hydrogel composites in deionized, tap, and saline water (0.5% and 1%), alongside the time‐dependent swelling behavior of hydrogels.

## 2 Results and Discussion

### 2.1 Characterization of Biochar and Biochar Hydrogel Composites

**Figure 1:**
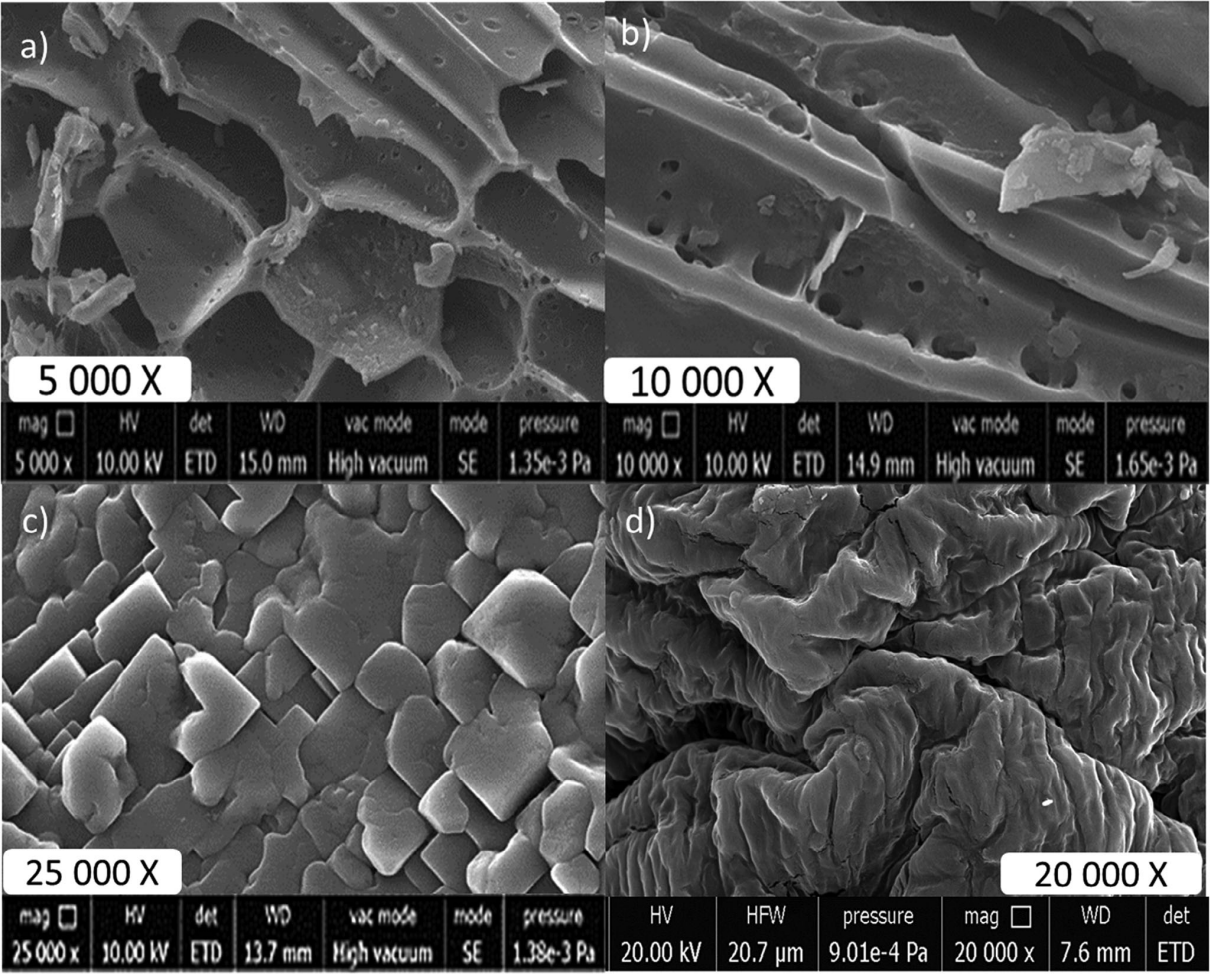
a) SEM images of biochar derived from vine (VB) magnification 5000X and b) magnification 10 000X, c) SEM images of VB doped hydrogels (VBHG), and d) Raw hydrogels.

In **Figure** **1**a–d, the surface morphologies of biochar hydrogel composites (VBHG) as well as biochar (VB) are depicted. SEM scans of the biochar samples revealed a noticeable variation between the samples. Different pore configurations, including micro, macro, and mesopores, were seen in the SEM micrographs of the generated VB. However, the VB biochars produced at 500 °C took on the appearance of a honeycomb with gaps in the cylindrical framework. Under a microscope, it can be seen that VB produces a large number of uniformly spaced pores. On the surfaces of VB, a regular pattern of tiny vertical blocks can be seen. Biochar exhibits an extremely porous structure, as observed in the SEM images. Specifically, the images in Figure 1a,b demonstrate that biochar particles possess a hollow, spherical, and well‐organized structure. These structures have thick walls that resemble a honeycomb pattern. The porous structure of biochar is formed during the pyrolysis or carbonization process, where biomass undergoes high‐temperature treatment under low‐oxygen conditions.^[^ ^32^ ^]^ During the thermal processing of biomass, organic matter evaporates, leaving behind a hollow structure as the volatile substances escape. This hollow structure allows biochar to have an extensive surface area. Biochar particles typically exhibit a certain regularity in their structure, forming an organized porous network between particles.^[^ ^33^ ^]^

SEM pictures of the hydrogels present in these biochar samples are shown in Figure 1c,d. Distinctive structures were observed in the SEM images of the hydrogels containing biochar (Figure 1c,d). The literature does not contain any examples of these SEM images that are similar. The observation that these structures exhibit brick‐like patterns and organized cubic structures is a noteworthy result. Cubic constructions in three dimensions frequently connect to one another. These cubic forms were not determined in the raw hydrogels (Figure 1d). Raw hydrogel surfaces revealed the architecture of wrinkles. The SEM images demonstrated how adding biochar to hydrogels affected their surface shape, and it was found that biochar was present not only within the hydrogels themselves but also on the surface. As a result of the hydrophilic group formations on the surface, this result affected the hydrogels' ability to swell.

**Figure 2:**
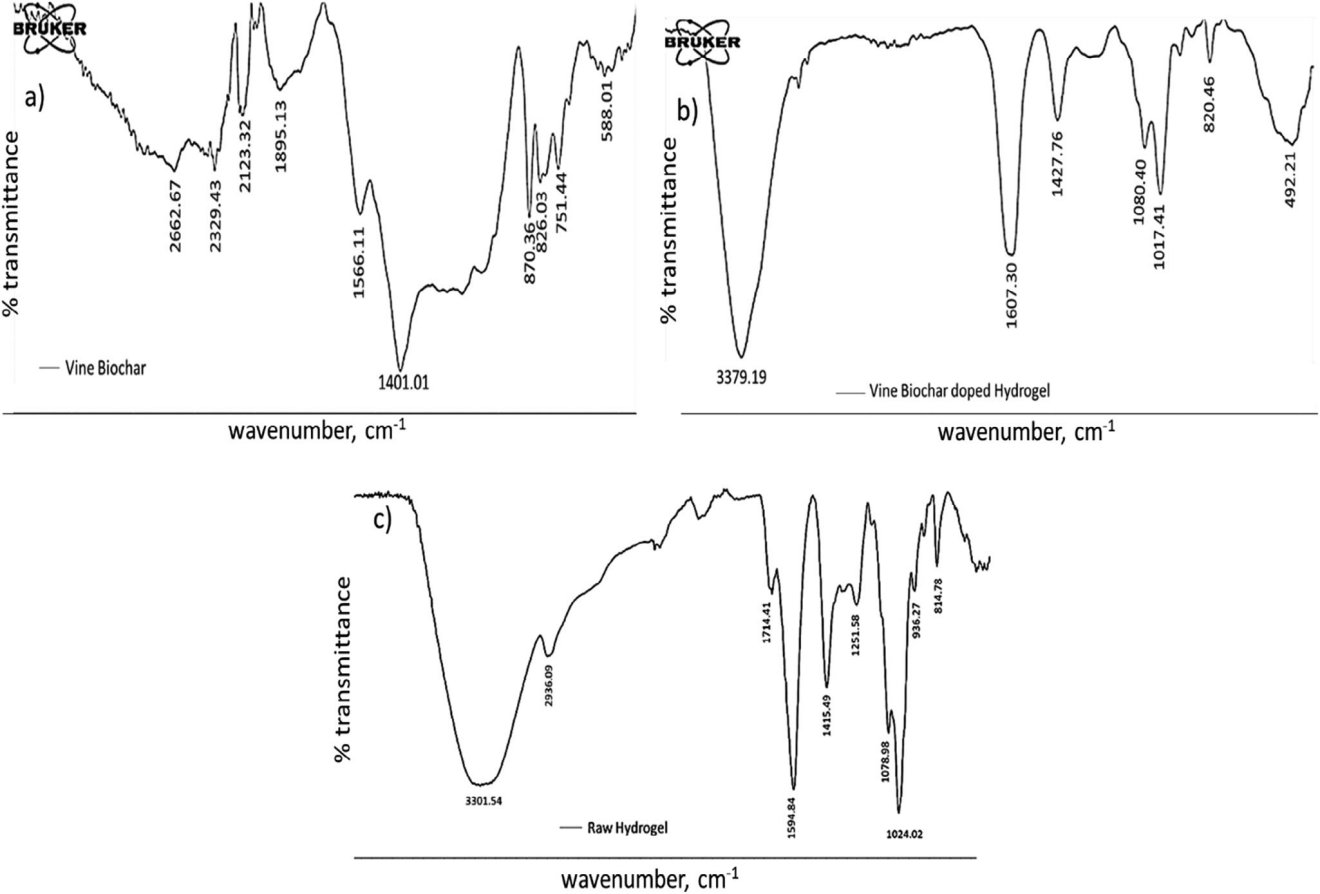
FT‐IR spectra of a) Vine biochar sample, b) Vine biochar doped hydrogel, and c) Raw hydrogel.

Biochar's FT‐IR spectra are displayed in **Figure** **2**a. **Table** **1** also provides more precise details about the chemical bonding, peak position, and intensity from Figure 2a. The majority of FT‐IR features in the analysis of the organic components of biochar are derived from organic functional groups. Due to heat degradation and a significant loss of oxygen atoms, a cyclic acid anhydride's vibration of C═O stretching is attributed to weak intensity ≈2100–2070 cm^−1^.^[^ ^34^ ^]^ According to Chen et al.,^[^ ^35^ ^]^ the aromatic group from lignin results in C═C asymmetric stretching at 1566 cm^−1^, which corresponds to the carbon atoms' sp^2−^hybridization bonding. In the VB, C─H bending mode was seen at 870 cm^−1^. According to Coates,^[^ ^36^ ^]^ Jipa et al.,^[^ ^37^ ^]^ and Siipola et al.,^[^ ^38^ ^]^ the methyl group (─CH_3_) stretching vibration is the cause of the transmittance at 1400 cm^−1^. According to Mary et al.,^[^ ^39^ ^]^ the presence of alcohol and aliphatic ether C═O is the cause of the transmittance at 1062 cm^−1^. Alkynes with C─H bending can be seen in the peak at 870–710 cm^−1^.^[^ ^40^ ^]^

**Table 1:** The functional groups in the VB and VB‐doped hydrogels.

VB	VBHG	Groups/Compound Class	Reference
–	3379	─OH stretching vibration band	[77]
2662	2919	C─H stretching for aliphatic (alkane/alkyl) groups	[42, 43, 44]
2104	–	C═O stretching	[34]
–	1607	C═O stretching	[45, 46]
1566	–	C═C stretching in the aromatic ring	[40]
1401	–	methyl group (─CH_3_) stretching vibration	[38]
–	1427	C═O stretching	[45]
1244	–	C═O stretching	[77]
1062	–	aliphatic ether C═O and alcohol	[39]
–	1080	C─O stretching (primary alcohol)	[48]
–	1017	C─O─C stretching bond from sodium alginate	[47]
870	–	C─H bending	[36]
826	820	aromatic C─H stretching vibration	[40]
–	492	C═O stretching bond from sodium alginate	[47]

The FT‐IR graphs in Figure 2b,c, as well as Table 1, provided confirmation of the most important functional groups in the VBHG composites and raw hydrogel samples. The FT‐IR analysis showed that the intermolecular interactions were what regulated the vibration of the functional groups on the superabsorbent hydrogel fragments. The vibration bands of ─OH stretching were responsible for the FT‐IR peaks at 3379 cm^−1^.^[^ ^36^, ^38^, ^41^ ^]^ The peak at 2919 cm^−1^, which was brought about by the C─H stretching vibrations of aliphatic groups, contained the ─CH_3_ and ─CH_2_ groups.^[^ ^42^, ^43^, ^44^ ^]^ According to Korbag and Saleh^[^ ^45^ ^]^ and Helmiyati and Aprilliza,^[^ ^46^ ^]^ the peak at 1607 cm^−1^ also demonstrated the C═O stretching of PVA. The many peaks in the region of 492–1427 cm^−1^ were shown by the FT‐IR peaks of biochar‐based hydrogels.^[^ ^36^, ^37^, ^38^ ^]^ The C═O stretching band indicated characteristics of both SA and PVA as a medium‐intensity peak at 1427 cm^−1^.^[^ ^45^ ^]^ A hydrogen connection can form between the hydroxyl groups of PVA and this SA group (raw hydrogel) since it is clear that when SA is added, the hydroxyl stretch bands broaden dramatically. The sodium alginate‐derived C─O─C stretching bond in the hydrogel is responsible for the peak at ≈1017 cm^−1^.^[^ ^47^ ^]^

According to Coates,^[^ ^36^ ^]^ Thayumanavan et al.^[^ ^48^ ^]^ and Ray et al.,^[^ ^40^ ^]^ the stretching vibrations of hydrogen‐bonding C─OH groups and aromatic C─H groups can be seen at 1080 and 820 cm^−1^ in FT‐IR spectra, respectively. At ≈490 cm^−1^, sodium alginate‐derived C═O stretching bonds were also visible.^[^ ^47^ ^]^ Also, the elemental composition of the produced VB was 72.9% C, 1.3% N, and 3.3% H, the pH value was 9.21 and the electrical conductivity was 995 µS cm^−1^.

### 2.2 Swelling Capacity of Hydrogels in Different Aqueous Media

**Figure 3:**
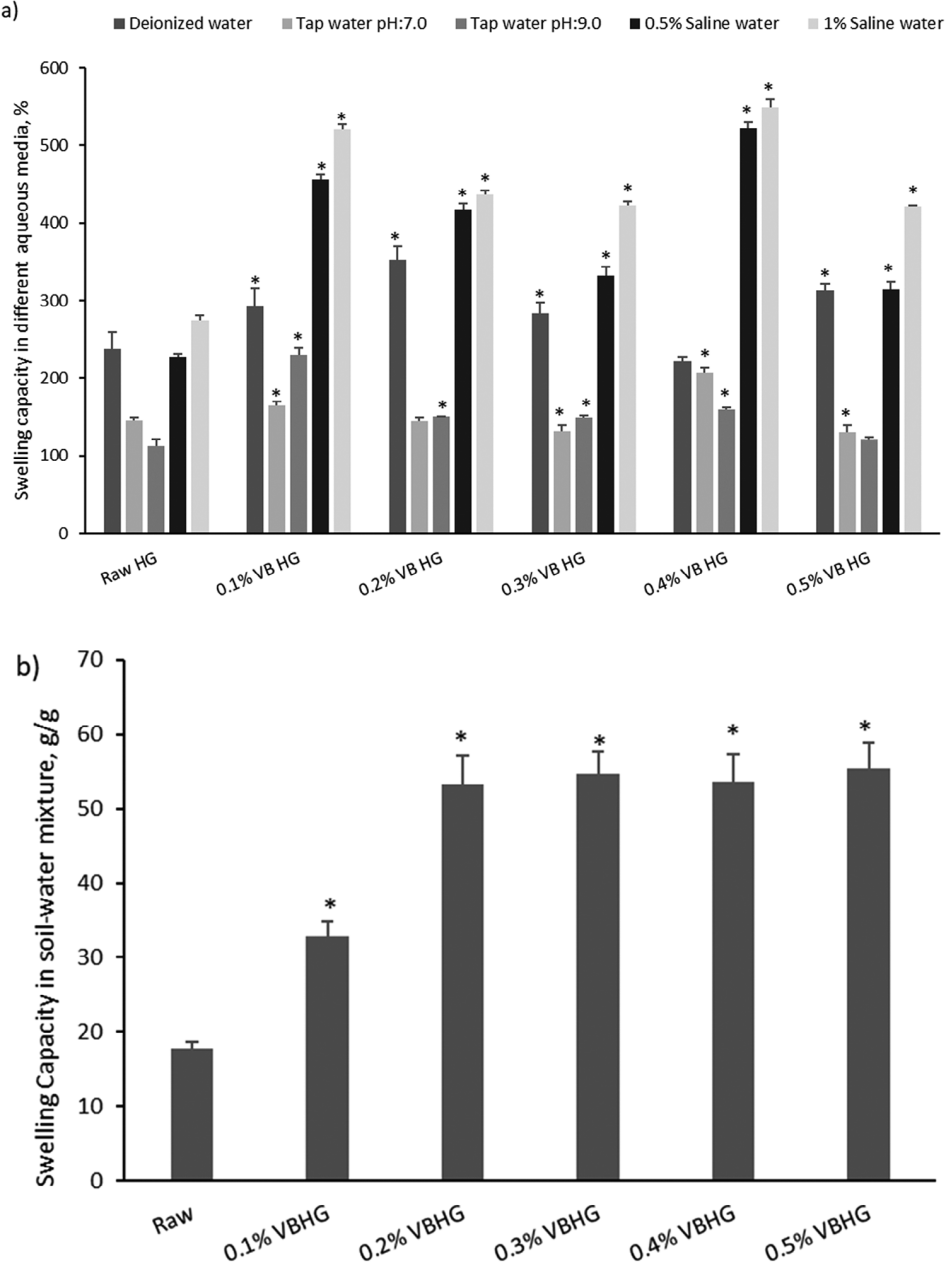
Swelling capacity of synthesized VBHG and Raw hydrogel a) different aqueous media (deionized water, tap water pH:7.0 and pH:9.0, and saline water 0.5% and 1%), b) soil‐water mixture (*means statistically significant compared to control, *p *< 0.05).

The swelling ratios of the synthesized Raw HG and VB‐doped hydrogels in different aqueous media (mentioned before) and in the soil‐water mixture were determined. The swelling properties of synthesized hydrogels depend on the amount of the biochar addition to hydrogels, as well as the swelling media. The maximum swelling capacity of VBHG was determined in deionized water, tap water (pH 7.0), tap water (pH 9.0), saline water (0.5%), and saline water (1%) as 352%, 207%, 230%, 522%, and 549%, respectively (**Figure** **3**a). The results showed that the biochar addition to hydrogels improved the swelling capacity, compared to Raw HG (*p* < 0.05). When the biochar amount in hydrogels was considered, 0.1 0.1% VB and 0.4% VB were suitable amounts. Besides that, the swelling capacity was enhanced in the saline media (0.5% and 1%). These water types can be found in nature. Thus, it is important to determine the swelling capacity of synthesized hydrogels, especially in agricultural applications. The swelling capacity of hydrogels in different aqueous media highly depends on the carboxyl groups in/on the hydrogel and the charge distribution on the surface of the hydrogel network (Gupta and Shivakumar, 2012;).^[^ ^49^ ^]^ The maximum swelling capacity was determined in the 0.1% saline media, due to the ionization of carboxylic groups (─COOH) and the relaxing of hydrogel bonds with increasing pH values.^[^ ^50^, ^51^ ^]^ This swelling mechanism can be explained by the salting‐in effect and penetration of water molecules to the hydrogel surface. Other mechanisms affecting the swelling capacity of hydrogels include capillary, osmotic pressure differences, and hydration forces, which are balanced by the forces between water molecules and polymers.^[^ ^44^, ^52^ ^]^ With the interaction between the water molecules and polymers, the polar groups in the polymer gain hydrophilic properties, and the hydrogels swell, while the physical and chemical strength properties of these polymers (such as cross‐links) preserve the 3D structure of the hydrogel.^[^ ^52^ ^]^

The hydrogels synthesized using vine pruning wastes biochar showed a good swelling capacity in the soil‐water mixture, compared to Raw HG (Figure 3b) (*p* < 0.05). Results proved that the biochar doping in hydrogels improved the swelling capacity. However, the swelling values in the soil‐water mixture remained below the swelling values in free water, as in the study of Duperkova et al.^[^ ^53^ ^]^ Although the maximum swelling capacity in the soil‐water mixture was determined at the content of 0.5% vine biochar in hydrogel as 55.37%, there were no significant differences between the other biochar content in hydrogels. The results of this study are consistent with other studies conducted by Abdallah.^[^ ^54^ ^]^

### 2.3 Time‐Dependent Swelling Behavior of Hydrogels

**Figure 4:**
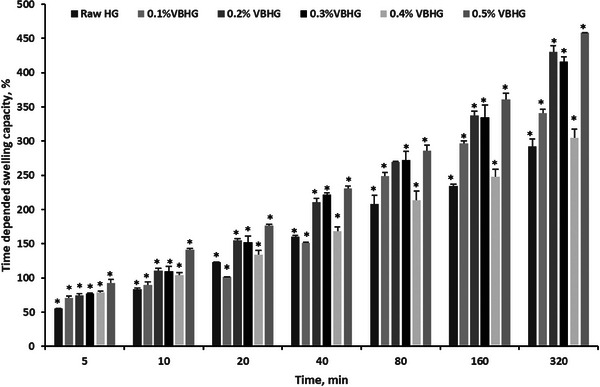
Time‐dependent swelling capacity of synthesized VBHG and Raw HG (*means statistically significant compared to control, *p* < 0.05).

To determine the swelling capacities at different time intervals, the experiments were conducted through 5.5 h. Results showed that the swelling capacity of synthesized Raw and biochar‐doped hydrogels increased with time (**Figure** **4**). In addition, the maximum swelling rate was found as 0.0024 g min^−1^ at 0.5% VB content in the hydrogel, while the minimum swelling rate was 0.0015 g min^−1^ at 0.4% VBHG. The minimum swelling capacity of hydrogel contained vine biochar was found in 0.4% VBHG as 305.2%, while the Raw HG swelled 291.9% after 320 min. In general, competition between water‐polymer and polymer‐polymer in raw hydrogels is primarily driven by water absorption, which results from the interaction between hydrogen bonds and water molecules, and which controls the swelling of hydrogels.^[^ ^50^ ^]^ In this study, it was determined that the swelling capacity of hydrogel was completely related to their complex structures which depend on the biochar amount. These complex structures formed between the strong hydrogen bonds from used polymers (PVA and SA) and the carboxyl groups of the biochar.^[^ ^34^ ^]^ This formation can change the swelling capacity of synthesized hydrogels. Results showed that the biochar content in hydrogels was the most effective parameter in the swelling capacity, swelling degree can be improved if well adjusted.^[^ ^55^, ^56^ ^]^ The swelling capacity can be sorted after 320 min as 0.5% (458.23%) >0.2% (430.13%) >0.3% (416.36%) >0.1% (341.38%) > 0.4% (305.2%) > Raw HG (291.87%), as like the other studies in the literature.^[^ ^57^, ^58^ ^]^

### 2.4 Swelling Curves and Diffusion Coefficients

**Figure 5:**
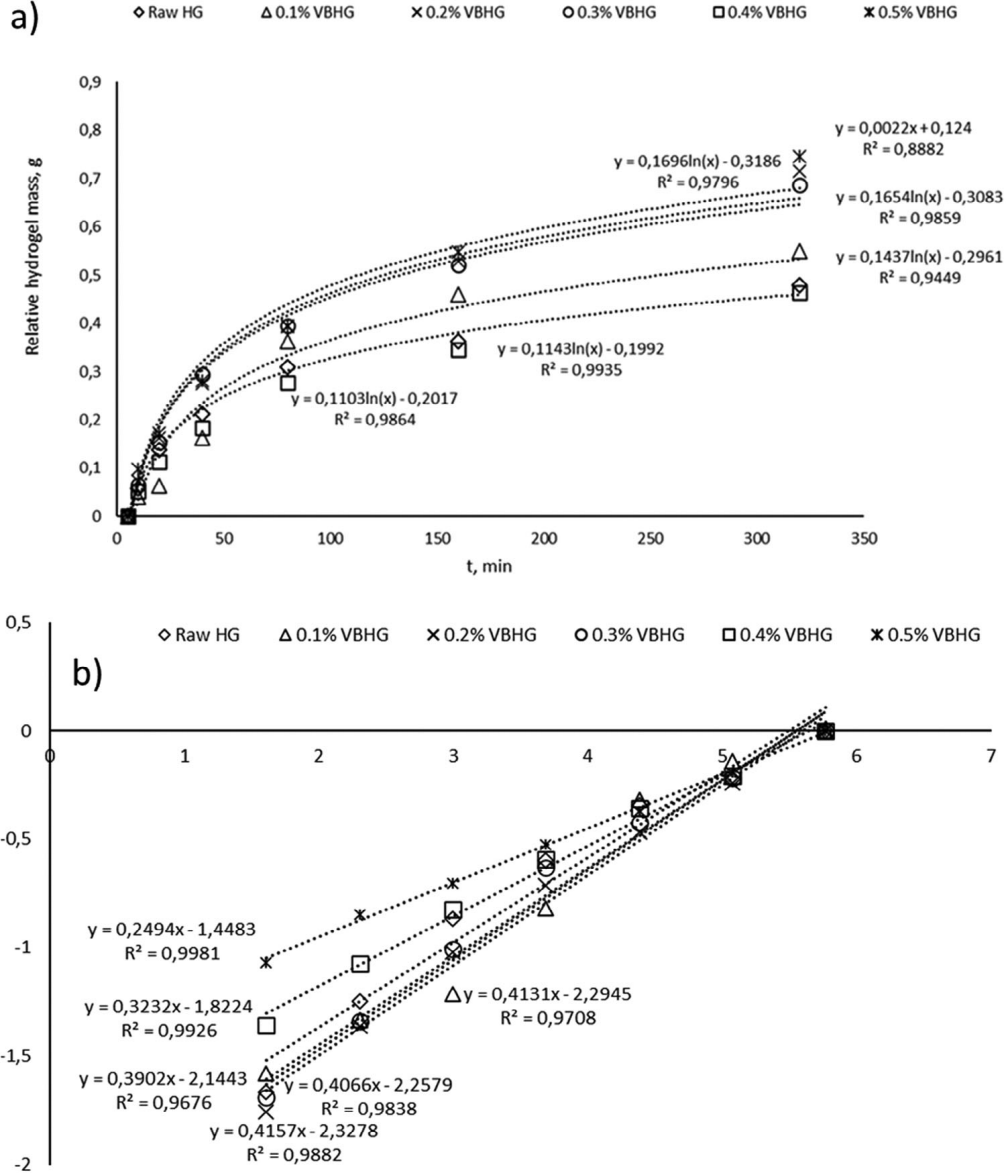
a) Change of the relative hydrogel masses with time and b) their swelling kinetic curves of VBHG.

Swelling is one of the key elements in the cross‐linked hydrogels' character analysis. At 25 °C ambient temperature, swelling experiments were conducted using the hydrogels of PVA/SA/BC composites. 100 mL of distilled water and 0.2 g of dried hydrogels were added to the beaker in order to measure swelling. The initial duration was set to zero (*t* = 0), and the hydrogel weights were determined after surface moisture measurements were taken at predefined intervals. The weights were then used to study the swelling kinetics. The hydrogel swellings reached equilibrium after roughly 300 min, according to the results. These data were used to produce the swelling kinetics (**Figure** **5**).

Fick's laws^[^ ^59^ ^]^ are the commonly used terms to describe water diffusion into the dried hydrogel and its diffusion type. The mechanics of water diffusion into the swelling hydrogel were examined using Equation (3). The plots of Ln(F) versus ln (t) were made using the logarithmic form of Equation (3), and these graphs may be seen in Figure 5. The values of n, k, r, and regression coefficients were calculated based on the slopes and intercepts of the plots of ln (F) against ln (t), respectively, and are shown in **Table** **2**. The kind of diffusion is determined by the value of n, which is the definition of Fick diffusion transport.^[^ ^60^ ^]^ The information from Equation (3) relates to the hydrogels' quick uptake of water diffusion. The time‐dependent swelling kinetics graphs in Figure 5 demonstrate how the swelling increases and the solvent mass enters the gel structure. For pure hydrogel without biochar, Fickian diffusion and Case II transport were reported to be 0.3902 and 0.2494–0.4157 for VBHG composite hydrogels containing 0.1–0.5% VB, respectively.

**Table 2:** Swelling coefficient values of raw HG and VBHG.

Coefficient	Raw HG	VBHG				
	0%	0.1%	0.2%	0.3%	0.4%	0.5%
r [cm]	0.333	0.417	0.400	0.317	0.333	0.400
n	0.390	0.413	0.416	0.407	0.323	0.249
k	1.477	1.511	1.515	1.501	1.381	1.283
R^2^	0.968	0.971	0.988	0.984	0.993	0.998
D	0.0271	0.0479	0.0304	0.0282	0.0130	0.0052

Fickian and Non‐Fickian Diffusion are two types of diffusion that are defined by Fick's laws. Contrary to non‐Fickian diffusion, Fickian diffusion abides with the Fickian laws. The main difference between Fickian and Non‐Fickian Diffusion is whether or not there are boundaries; although Fickian Diffusion has no limits, Non‐Fickian Diffusion has a firm barrier dividing the highly inflated zone from a dry, glassy region. This theory predicts that in some polymer systems, the boundaries between the swelled and non‐swollen regions are distinct and change linearly over time. These swelling exponents (n) showed that the Non‐Fickian diffusion character was engaged in the transport of all superabsorbent polymers. The values for k varied between 1.283–1.515 for VBHG. This value was obtained as 1.477 for Raw HG. The high regression coefficient values (R^2^) were obtained for all biochar‐doped hydrogel (BHG) composites in the range of 0.9676–0.9981. The diffusion coefficient (D), which is calculated using Equation (4), is one of the crucial parameters used to describe the swelling of the hydrogel. Table 2 contains the hydrogels’ determined values for the diffusion coefficient. The diffusion coefficient (D value) for Raw HG was obtained as 0.0271. The VBHG samples showed a highly variable D value depending on the % of biochar they contained. The lowest D value was obtained as 0.052 in the vine hydrogels containing 0.5% BC, while the highest D value (0.0479) was reached in the 0.1% sample containing the lowest BC.

### 2.5 Re‐Swelling Properties

**Figure 6:**
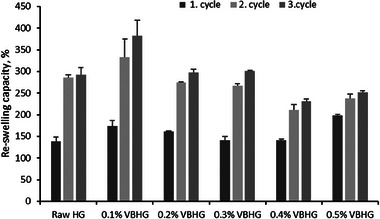
Re‐swelling capacity of synthesized VBHG and Raw HG after the third cycle.

It is important to determine the re‐swelling capacity of hydrogels in agricultural applications due to the adjustment of the irrigation period. **Figure** **6** shows three swelling/drying cycles of synthesized hydrogels. Results showed that the synthesized Raw and VB‐doped hydrogels continued to noticeably swell in the third cycle. The re‐swelling properties depend on the hydrogel structure (i.e., elasticity, crosslink density, etc.).^[^ ^61^ ^]^ Besides that, the biochar in hydrogels has a key role in the re‐swelling capacity, due to the co‐existence of many functional groups such as S═O, C═O, COOH, and C─OH in its structure.^[^ ^56^ ^]^ The results observed in the swelling/drying cycles showed that the amount of biochar content of the hydrogels is not affected by the swelling behavior, and the increase or decrease in the swelling capacity of the hydrogel may be due to the deterioration of the polymeric structure. The maximum and the minimum re‐swelling capacity were determined at 0.1% VBHG (381.9%) and 0.4% VBHG (231.3%) after the third cycle, respectively. However, these values were not statistically significant, when compared to Raw HG (*p* > 0.05). The synthesized hydrogels still maintained ≈200% swelling capacity even after three times, this result showed that the synthesized hydrogels had excellent water absorption performance. The re‐swelling mechanisms and effective parameters were given in our previous studies,^[^ ^62^ ^]^ also the results fit with our previous studies,^[^ ^62^, ^63^ ^]^ and the other studies in the literature.^[^ ^64^, ^65^, ^66^, ^67^ ^]^

### 2.6 Seed Germination Tests

**Figure 7:**
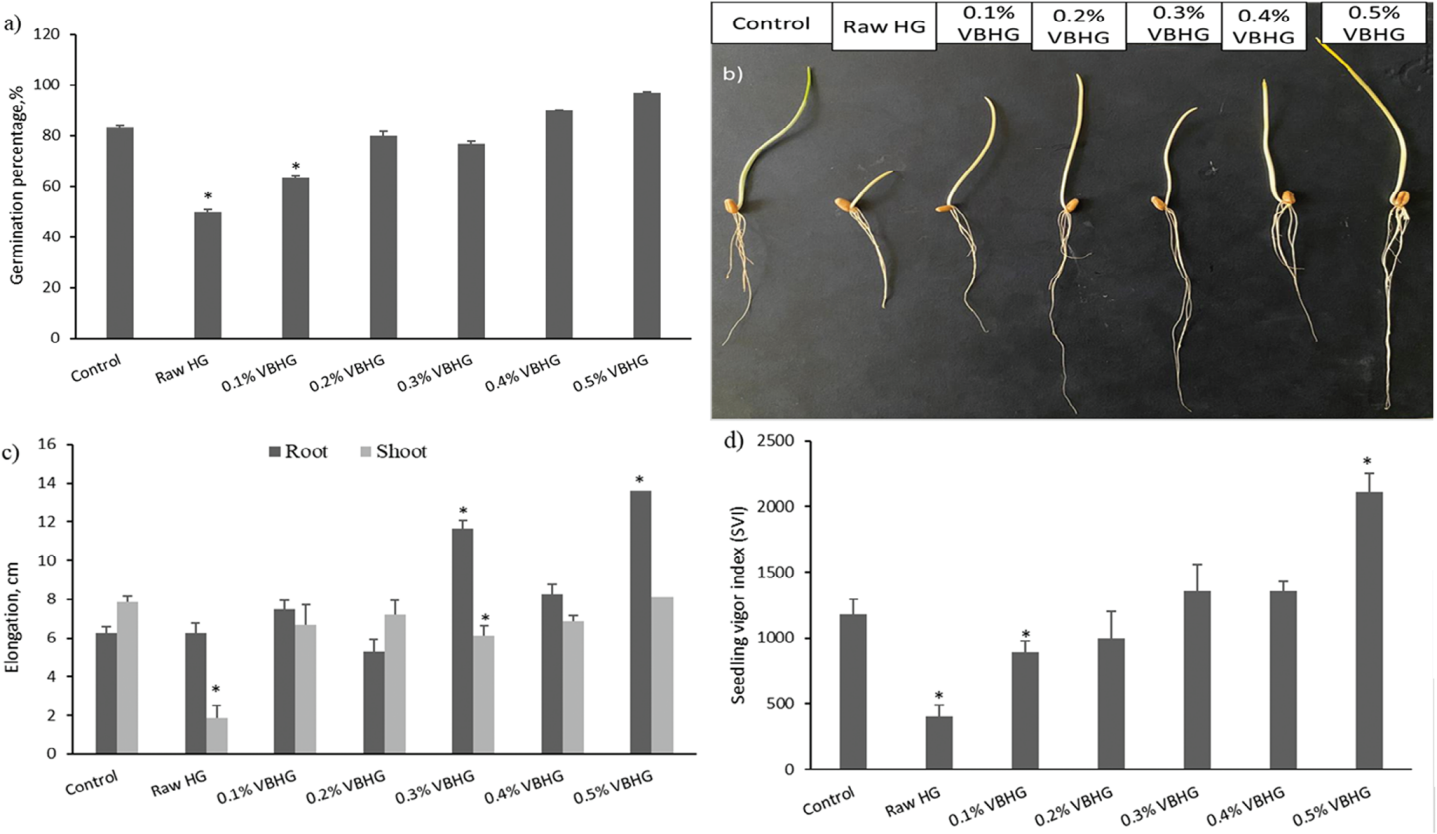
a) Germination percentage of wheat seeds under treatment of VBHG and Raw HG, b) image of seedlings after germination, c) root and shoot elongation of wheat seedlings, and d) seedling vigor index of wheat (*means statistically significant compared to control, *p* < 0.05).

In this study, the germination experiments were conducted to determine the effects of synthesized hydrogels in the seed germination. Results indicated that the presence of biochar in synthesized hydrogels did not significantly affect the seed germination of wheat, compared to control (**Figure** **7**a), except 0.1% (*p* > 0.05). However, it is noteworthy that the presence of biochar in the hydrogels ensures the elimination of the limiting effect caused by the raw hydrogel. It is estimated that the C and N content supported the germination process of wheat seed. This result was in a similar tend to Cao and Li.^[^ ^68^ ^]^ The author showed that the synthesized agarose hydrogel limited the rapeseed germination until the 7th day. However, when the agarose and activated carbon together in hydrogel synthesized, the germination percentage reached 100%, especially at the content of agarose: activated carbon rate as 10:3.^[^ ^68^ ^]^ The VBHG positively affected the wheat seed germination compared to raw hydrogels. The highest germination percentages were determined at the treatment of 0.4% and 0.5% VBHG as 90% and 97%, respectively. The germination results were parallel with the literature about the effect of hydrogels on different plant seeds.^[^ ^61^, ^66^, ^69^ ^]^ After the germination process (7 days), the root and shoot elongation of wheat seedlings was determined to see clearly if there were any phytotoxicological effects of Raw and VB‐doped hydrogels. The highest root elongation was determined at 0.3% and 0.5% VBHG treatment as 11.7 and 13.6 cm, respectively (*p* < 0.05) (Figure 7b). The other hydrogel treatments positively affected the root length of wheat seedlings, but it was not statistically significant. Besides that, the shoot elongation decreased compared to the control, and the changes were not statistically significant, too. The minimum shoot length was measured at the treatment of Raw HG as 1.9 cm (*p* < 0.05). Qin et al.^[^ ^70^ ^]^ showed those cellulose‐based hydrogels have a positive effect on root elongation of wheat, compared to Raw HG, like Cao and Li^[^ ^68^ ^]^ who used agar‐activated carbon‐based hydrogels. Based on our experience, it can be said that biochar‐doped hydrogels positively affect plant growth, due to their well‐elemental content (C, N, H) (^[^ ^62^, ^63^ ^]^; Doğaroğlu 2023). The elemental composition (C, N, H) of vine pruning wastes biochar mentioned before.

The seedling vigor index (SVI) depends on the seed and seedling performance, and it can show whether the seedling is under stress or not. Figure 7d shows the SVI values increased with the treatments of VBHG, compared to the control. The maximum and the minimum SVI values were determined as 2105 in 0.5% VBHG treatment and 407 in Raw HG treatment, respectively. This result supported that biochar‐doped hydrogel enhanced the seed germination compared to Raw HG and root‐shoot elongation compared to control and Raw HG (Figure 7d).

## 3 Conclusion

In this study, cellulose‐containing agricultural waste (vine pruning wastes) was used to make biochar, and a hydrogel composite (VBHG) containing the biochar was made. The characterization procedures of synthesized biochar (VB), biochar doped (VBHG), and raw hydrogels, were carried out using techniques FT‐IR and SEM. The maximum swelling capacity of the biochar‐doped hydrogels was determined in the 1% saline media due to the salting‐in effect and the absorption mechanisms. The time‐dependent behavior and polymers' swelling dynamics showed that the maximum swelling capacity was found at the treatment of 0.5% VBHG (458.23%) due to the functional groups in/on HG, and water transportation mechanism was a non‐Fickian diffusion character. Re‐swelling property showed that the synthesized hydrogels were continuously swelling after the third drying/swelling cycle. The germination result showed that there were not any significantly toxic effects of synthesized VBHG. Hydrogel can be used as an alternative seed germination medium. The maximum increase in root elongation was calculated as 217.6% at the treatment of 0.5% VBHG. The maximum decrease in shoot elongation was calculated as 23.8% at the treatment of Raw HG. All these hydrogel applications are not only effective in drought‐related studies, but also can be considered as a promising application in soilless agricultural applications.

## 4 Experimental Section

### Production of the Biochar and Chemical Examinations of the Materials

The biomass of vine pruning wastes (VB) (5 cm) was oven‐dried at 65 °C for 48 h before being pyrolyzed using a pyrolysis reactor (UnitermLab) at a high temperature utilizing a gradual pyrolysis method (500 °C hold temperature, 4 h, and 10 °C min^−1^ heat rate). The produced biochar was removed from the reactor and stored in a desiccator until it reached room temperature. They were crushed, sieved (0.2 mm), and kept for additional examinations. Eurovector's EA3000‐Single Analyzer performed elemental analyses of the created biochars to assess their C, H, and N content percentages. After treatment with 0.01 m CaCl_2_ (w/v = 1/5), the pH values of the biochar samples were evaluated using a pH meter (Thermo Scientific, Orion Star‐A111). According to Das and Ghosh,^[^ ^34^ ^]^ the electrical conductivity values (EC) were assessed using a multi‐parameter (Hach, HQ440d) following filtration of the biochar/deionized water solution (w/w = 1/10).

### Synthesis of Biochar‐Doped Hydrogels

The pure hydrogel beads were made according to Putra and Lee.^[^ ^71^ ^]^ PVA (5 g) and SA (5 g) were combined and autoclaved in 100 mL of deionized water for 30 min at 120 °C. The solutions were then added slowly, using a syringe, to a solution of CaCl_2_ (5% w/v), which was agitated at 100 rpm. The solutions were left in the CaCl_2_ solution overnight to complete the cross‐linking process. A varied amount of each of the three types of biochars (0.1‐0.2‐0.3‐0.4‐0.5%, w/v) was introduced into the solution and properly mixed in order to produce biochar‐doped composite hydrogels following autoclaving. As pure hydrogel beads, homogenous drops of the BC/PVA/SA solution were dropped into CaCl_2_. To eliminate any remaining CaCl_2_, deionized water was used to wash the produced pure and BC/PVA/SA hydrogel beads.

### Characterization of BCs and BC Composite Hydrogels (VBHG)

SEM (Quanta 650 FEG made by FEI, USA) was used to examine the surface morphologies of the VB, VBHG, and raw hydrogel beads. FTIR spectra (FT/IR‐6700, Jasco) were used in KBr pellet procedures to investigate the materials' chemical bonding in the 4000–500 cm^−1^ range.

### Swelling Capacity of Hydrogels in Aqueous and Soil Media

According to Feng et al.,^[^ ^27^ ^]^ the ability of synthesized hydrogels to swell in aqueous media was determined. In order to do this, synthesized hydrogel beads were dried for 2 days at room temperature (25 °C) and left in an oven at 80 °C for an hour to finish the dehydration process. In order to reach the equilibrium swelling degree, 0.2 g of dried hydrogels (w_dry_) were placed in distilled water, tap water (pH 7.68 and 9.0), and saline water (0.5% and 1%) at room temperature for 4 days. After being taken out of the water and briefly dried using wet filter paper, the swollen hydrogel beads were instantly weighed (w_swelled_). There were three replicates of each treatment run. Using Equation (1), the equilibrium swelling ratio was calculated:

(1) $$\mathrm{ESR}\;\left(\%\right)=\frac{\left(W_{\mathrm{swelled}}-W_{\mathrm{dry}}\right)}{W_{\mathrm{dry}}}\;\times 100$$

The experiments were carried out in accordance with the methodology outlined by Abdallah^[^ ^54^ ^]^ to estimate the swelling capacity (SC) in soil media. The 100 g samples of sandy soil, 1 g of hydrogels, and 1 g of hydrogel plus 100 g of loamy sand soil were weighed and transported in permeable nylon bags to assess the swelling capacity of hydrogels with different types and amounts of biochar in free water and the water‐soil mixture. The bags were then submerged for 15 min in a glass beaker containing 2 L of tap water (pH 7.0). The gravimetric measurement of the hydrogels' swelling potential in both free water and a water‐soil mixture allowed for the following calculation (Equation 2). There were three replicates of each treatment run.

(2) $$SC\;=\;\frac{\left(W_{\mathrm{wet}\;\mathrm{hydrogel}\;+\;\mathrm{soil}}\;-\;W_{\mathrm{wet}\;\mathrm{soil}}\right)}{W_{\mathrm{dry}\;\mathrm{hydrogel}\;\mathrm{in}\;\mathrm{mixture}}}$$

The soil texture used in this study was loamy sand and the other properties were as pH:7.37, electrical conductivity: 628 µs cm^−1^, organic matter content 1.21%, lime 24%, clay (<0.002 mm) 1.3%, silt (0.002–0.06 mm) 10%, sand (0.06–2 mm) 88%, saturation degree 33%, and salt 0.012 dS m^−1^.

### Time‐Dependent Swelling Behavior of Hydrogels

According to Liu and Huang,^[^ ^72^ ^]^ the swelling kinetic studies of raw and biochar composite hydrogels were carried out. In order to do this, 0.2 g of dried composite hydrogels were placed in distilled water and weighed at intervals of 5–10–20–40–80–160–320 min at room temperature. The hydrogels were taken out of the water for the first measurement after 5 min, slightly dried with wet filter paper, and weighed right away. They were then put back into the water. This process was continued for an additional minute, and Equation (1) was used to compute the swelling kinetics at each interval of time. There were three replicates of each treatment run.

### Water Diffusion Model of Hydrogels

Fick's most fundamental law is used to describe swelling kinetics and polymeric structure diffusion. The following (Equation 3) is a calculation of the dynamics of polymer swelling using the Fick equation^[^ ^73^, ^74^ ^]^:

(3) $$F\;=\frac{S_{t}}{S_{e}}\;=\;kt^{n}$$

where “F” stands for the swelling fraction, “Se” is for the equilibrium swelling level of the hydrogel, “k” is for the solvent's diffusion exponential, and “c” is for the constant that varies depending on the network topology of the gel. To identify the kind of diffusion, the value “n” must be known. The “n” diffusion exponent is estimated using the slope of the lines created by linearizing the data from the determined F values (swell fraction) on the lnF‐lnt plots. A significant factor in the investigation of swelling kinetics is the coefficient of diffusion. The equation^[^ ^75^ ^]^ that arises from the arrangement of Fick's II law (Equation 4) can be used to compute the coefficient of “D” for cylindrical objects.

(4) $$D\;=\;\pi r^{2}{\left(k/4\right)}^{1/n}$$

where “D” represents the coefficient of diffusion as “m^2^ s^−1″^ and “r” represents the swollen gels radius as “m.”

### Re‐Swelling Capacity of Hydrogels

The dried hydrogels were placed in deionized water for 80 min then they were slightly dried using wet filter paper and weighed. The weighed‐swelled hydrogels were left in an oven at 80 °C to dry. After that, they were left in deionized water again for 80 min, they were weighed and dried at 80 °C again. Three repetitions and three copies of this cycle were performed.^[^ ^54^ ^]^

### Seed Germination Experiments

Seed germination studies were carried out to ascertain the phytotoxicological effects of produced hydrogels on wheat according to Doğaroğlu et al.^[^ ^76^ ^]^ The wheat seeds utilized in this investigation were bought in Mersin, Turkey. The wheat seeds (*Triticum aestivum* L. ikizce‐96) were undoubtedly sterilized using 70% ethanol and 5% sodium hypochlorite solution, respectively, and then rinsed five times for 5 min with deionized water. Ten uniformly sized wheat seeds were put in Petri dishes (100 × 20 mm) after being sterilized on two layers of filter paper. After that, seeds were covered with 2 g of raw, biochar‐doped hydrogels and 3 mL of deionized water in Petri plates. The hydrogels served as a source of water for the germination of the seeds. The hydrogels were not introduced to the Petri plates for the control groups, and only 5 mL of deionized water was used for the germination procedure. For 7 days, the Petri dishes were kept in the dark at 25 °C. Three replicas of each treatment were used in the experiment. The number of germinated seeds (radicle length>5 mm) was counted after the germination process (7 days), and the germination percentage (%) was computed (Equation 5).^[^ ^76^ ^]^ The seedling vigor index (SVI) was also determined based on the germination % and average root and shoot length. (Equation 6).

(5) $$\begin{array}{cc} & \mathrm{Germination}\;\mathrm{Percentage}\;\;\left(\mathrm{GP}\right)\\ & \;=\frac{\mathrm{Total}\;\mathrm{number}\;\mathrm{of}\;\mathrm{germinated}\;\mathrm{seeds}}{\mathrm{Total}\;\mathrm{number}\;\mathrm{of}\;\mathrm{tested}\;\mathrm{seeds}}\;\times 100 \end{array}$$

(6) $$\begin{array}{ccc} & & \mathrm{Seedling}\;\mathrm{Vigor}\;\mathrm{Index}\;\;\left(\mathrm{SVI}\right)\\ & & =\;\mathrm{GP}\left(\%\right)\times \;\left(\mathrm{Root}\;\mathrm{lenght}+\mathrm{Shoot}\;\mathrm{Lenght}\right) \end{array}$$

### Statistical Analyses

The results were statistically evaluated using the ANOVA with LSD test. Version 20 of the SPSS Statistic was used to carry out the calculations. The significance of the difference for each measurement was determined using the least significant difference, or LSD, with a *p*‐value of 0.05.

## Conflict of Interest

The authors declare no conflict of interest.

## Data Availability

The data that support the findings of this study are available from the corresponding author upon reasonable request.
